# Hepatopulmonary syndrome in children and adolescents with portal hypertension in Brazil: A multicenter study

**DOI:** 10.1002/jpn3.70306

**Published:** 2025-12-05

**Authors:** Leticia Drumond Alberto, Eleonora Druve Tavares Fagundes, Adriana Teixeira Rodrigues, Thaís Costa Nascentes Queiroz, Gustavo Valverde de Castro, Carlos Oscar Kieling, Sandra Maria Gonçalves Vieira, Natália Lamounier dos Martires Guerra, Natascha Silva Sandy, Gilda Porta, Irene Kazue Miura, Maria Ângela Bellomo Brandão, Gabriel Hessel, Adriana Maria Alves de Tommaso, Alexandre Rodrigues Ferreira

**Affiliations:** ^1^ Hospital das Clínicas da Universidade Federal de Minas Gerais Belo Horizonte Brazil; ^2^ Medical School of Universidade Federal de Minas Gerais Belo Horizonte Brazil; ^3^ Hospital de Clínicas de Porto Alegre Porto Alegre Brazil; ^4^ Hospital das Clínicas da Universidade de São Paulo São Paulo Brazil; ^5^ Hospital Infantil Menino Jesus São Paulo Brazil; ^6^ Hospital das Clínicas da Universidade Estadual de Campinas Campinas Brazil

**Keywords:** hypoxemia, liver cirrhosis, liver transplantation

## Abstract

**Objectives:**

To describe the clinical and laboratory characteristics and outcomes of pediatric hepatopulmonary syndrome (HPS) secondary to portal hypertension (PH) in Brazil.

**Methods:**

Fifty‐four pediatric patients diagnosed with PH and HPS according to the European Respiratory Society criteria were included in this multicenter retrospective study. Clinical and laboratory data at the time of diagnosing the underlying disease, 12 months before diagnosing HPS, at the time of diagnosing HPS, and at the time of the last consultation were collected from the medical records.

**Results:**

PH was cirrhotic in 87% of patients. Biliary atresia was the predominant etiology (35.2%). The median age at the time of diagnosis was 7.8 years (interquartile range [IQR]: 4.7–10.8). Partial arterial oxygen pressure (PaO_2_) of asymptomatic patients (44.4%) was higher than that of symptomatic patients (*p* < 0.0001). Peripheral oxygen saturation (SpO_2_) was ≥96% in eight patients, seven of whom had PaO_2_ of <70 mmHg. The hemoglobin levels were elevated at the time of diagnosing HPS (*p* = 0.009), whereas the platelet count was decreased (*p* < 0.001). The survival rates of the 66.6% of patients who underwent liver transplantation (LT) at 2 months and 1 year post‐LT were 90.3% and 84.6%, respectively. The severity of HPS did not affect the general post‐LT survival (*p* = 0.787).

**Conclusions:**

HPS remains asymptomatic during initial stages. SpO_2_ may not be a reliable screening test in pediatric patients. Elevated hemoglobin levels in PH may be related to hypoxemia. The severity of hypoxemia at the time of diagnosis does not affect post‐LT survival.

## Abstract (highlights)

**What is Known:**

- The hepatopulmonary syndrome (HPS) is a portal hypertension (PH) complication that affects cirrhotic and non‐cirrhotic pediatric patients. Its prevalence is higher in patients with cirrhosis.
- Predictive factors for the development of HPS remain to be established.

**What is New:**

- Elevated hemoglobin levels in patients with PH and hypersplenism may indicate the onset of hypoxemia and HPS.
- The severity of hypoxemia at the time of diagnosing HPS did not affect the post‐liver transplantation survival rates.

## 1 INTRODUCTION

Hepatopulmonary syndrome (HPS), a complication of portal hypertension (PH) observed in patients with and without cirrhosis, can be attributed to the high circulating levels of vasodilators and angiogenic factors inducing microvascular remodeling in the pulmonary territory, with consequent alteration of the ventilation/perfusion (V/Q) ratio and the onset of progressive hypoxemia. Liver transplantation (LT) remains the only definitive treatment option for HPS.^1^

In 2016, the International Liver Transplantation Society (ILTS) reaffirmed the following diagnostic criteria set forth by the European Respiratory Society (ERS) in 2004: (1) V/Q disturbance of ≥15 mmHg (or ≥20 mmHg if age is >64 years) documented using the alveolar‐arterial oxygen (A‐aO_2_) gradient in arterial blood gases (ABG) while breathing room air; (2) intrapulmonary vascular dilations (IPVD) documented using contrast enhanced transthoracic echocardiography (CE‐TTE) or ^99m^Technetium‐labeled albumin macroaggregates lung perfusion scintigraphy (^99m^Tc‐MAA); (3) underlying diagnosis of liver disease, PH, or congenital portosystemic shunts.^2^, ^3^

Although considered rare initially, the adoption of the ERS/ILTS diagnostic criteria and the performance of routine arterial blood gas analysis in some LT services has revealed a prevalence rate of >40% in children.^4^, ^5^, ^6^, ^7^ Nevertheless, most pediatric case series comprised only a few patients, resulting in gaps in knowledge regarding prognostic factors and related outcomes.^1^

Thus, this study aimed to describe the clinical and laboratory characteristics of Brazilian children and adolescents with HPS associated with PH to provide insights into this condition in pediatric patients.

## 2 METHODS

This retrospective observational multicenter study included patients from five reference centers for children with chronic liver disease (CLD) and PH across Brazil: Hospital das Clínicas of the Federal University of Minas Gerais (UFMG), Hospital de Clínicas of Porto Alegre, Hospital das Clínicas of the University of São Paulo, Hospital Sírio Libanês‐Hospital Menino Jesus of São Paulo, and Hospital das Clínicas of the University of Campinas.

Patients aged <18 years diagnosed with CLD or noncirrhotic PH with HPS diagnosed according to the ERS/ILTS criteria (A‐aO_2_ gradient ≥15 mmHg in ABG while breathing room air and IPVD documented by CE‐TTE or ^99m^Tc‐MAA) from 1997 to 2023 at the participating centers were included in the present study.^2^, ^3^ Patients with pulmonary or cardiovascular comorbidities and those whose confirmatory test results were unavailable were excluded. Diagnoses were made based on clinical suspicion or on ABG measurements obtained during pre–LT assessments, as ABG analysis is not routinely performed for all children with CLD in our centers.

Relevant demographic and clinical characteristics, examination results, blood biochemistry studies, hemogram and arterial blood gas analysis results were collected from patients' medical records at the following time‐points: at the time of diagnosing the underlying disease, 12 months before diagnosing HPS, at the time of diagnosing HPS, and at the time of the last consultation at the reference service. In addition, data regarding the indications, performance, and evolution of LT were also collected. Data between the cirrhotic and noncirrhotic groups were compared as well as data from cirrhotic patients due to biliary atresia (BA) and other CLD. A paired analysis was conducted with patients' laboratory data over time.

The arterial oxygen (PaO_2_) was used to classify the severity of HPS as mild (PaO_2_ ≥ 80 mmHg), moderate (PaO_2_ 60–79 mmHg), severe (PaO_2_ 50–59 mmHg), and very severe (PaO_2_ < 50 mmHg).^3^ The hyperoxia test was defined as the PaO2 measured while breathing room air and subsequently measured after breathing 100% oxygen. Oxygen therapy was defined as the need for home oxygen supplementation due to hypoxemia, maintained until LT, death, or the last follow‐up. The follow‐up duration was defined as the interval between the diagnosis of HPS and the last consultation at the reference service, with or without LT.

### 2.1 Ethics statement

This study was registered in the Brazilian National Database for research involving human subjects (http://plataformabrasil.saude.gov.br/login.jsf, reference number: CAAE 44356821.7.0000.5149) and approved by the Institutional Review Board of UFMG and collaborating centers. Informed consent and assent forms were obtained from parents/guardians and children/adolescents, respectively.

### 2.2 Statistical analysis

Research Electronic Data Capture (REDCap) digital platform was used to construct a database.

Descriptive measures are presented as median (Q2), quartiles (Q1 and Q3), mean, and standard deviation (SD) to describe quantitative variables. Descriptive measures are presented as absolute (*n*) and relative (%) frequencies to describe categorical/ordinal variables. Quantitative variables were compared between the two independent groups using the nonparametric Mann–Whitney *U*‐test. Quantitative variables were compared between ≥3 independent groups using the Kruskal–Wallis test. Quantitative variables measured at different times in the same individuals (dependent samples) were compared using the Friedman test. Multiple comparisons between different periods were performed using the Bonferroni correction. The association between two categorical variables was assessed using Pearson's chi‐square and Fisher's exact tests. Survival analysis was conducted using the Kaplan–Meier curve and log‐rank test. Statistical significance was set at <5%. The effect size was calculated using non‐parametric tests to assess the magnitude of the observed results, regardless of the sample size. All statistical analyses were conducted using SPSS 23.0 (IBM Corp.).

## 3 RESULTS

### 3.1 General data and diagnosis of the underlying disease

This study included 54 patients (female patients, 57.4%). PH was associated with cirrhosis in 87% of cases. The etiologies of cirrhosis were BA, cryptogenic cirrhosis, autoimmune hepatitis (AIH), primary sclerosing cholangitis, inborn error of metabolism, secondary sclerosing cholangitis, progressive familial intrahepatic cholestasis (PFIC), and Caroli syndrome in 35.2%, 22.2%, 7.4%, 7.4%, 5.6%, 3.7%, 3.7%, and 1.8% of patients, respectively. The noncirrhotic patients had extrahepatic portal vein obstruction (EHPVO) (9.3%) and congenital hepatic fibrosis (3.7%). The median age at the time of diagnosing PH was 2.5 years (1.1–7.3).

Esophageal varices were observed in 41 of the 46 patients who underwent endoscopic examination, with upper gastrointestinal bleeding episodes being reported in 44.4% of patients. Child‐Pugh classification (*n* = 46) was A, B, and C in 54.3%, 32.6%, and 13% of patients, respectively. The median pediatric end‐stage liver disease (PELD) and model for end‐stage liver disease (MELD) scores (*n* = 35 and *n* = 10, respectively) were 1 (−3.0 to 9.0) and 9.5 (4.3–11.3), respectively.

### 3.2 HPS diagnosis

The median age at the time of diagnosing HPS was 7.8 years (4.7–10.8) and the median interval between the diagnosis of PH and that of HPS was 4 years (2.3–6.3).

Arterial blood gas analysis revealed that the mean PaO_2_ was 58.2 ± 10.9 mmHg in all patients diagnosed with V/Q disorder. HPS was classified as moderate (44.5%), severe (37%), and very severe (18.5%). IPVD was confirmed through CE‐TTE and ^99m^Tc‐MAA in 98.1% and 40.7% of patients, respectively. Among the 25 patients who underwent a hyperoxia test, 4 and 10 patients had a PaO_2_ of >300 mmHg and ≤150 mmHg after hyperoxia, respectively.

At the time of diagnosing HPS, 44.4% of patients were asymptomatic. The remaining patients presented with dyspnea. The mean PaO_2_ of the asymptomatic patients was significantly higher than that of those with dyspnea (64.9 vs. 52.9 mmHg, *p* < 0.0001). One patient (1.9%) presented with platypnea, the most specific symptom of HPS. The mean peripheral oxygen saturation (SpO_2_) of patients at the time of diagnosing HPS was 88.4 ± 8.9%. Eight patients (14.8%) with a mean PaO_2_ of 58.4 ± 9.8 mmHg had the SpO_2_ ≥ 96%. One of those had a PaO_2_ of 79.5 mmHg.

### 3.3 Comparison between cirrhotic and noncirrhotic patients and between those with BA and other CLD

Comparison between the clinical and laboratory characteristics of the cirrhotic and noncirrhotic groups revealed no statistically significant differences (Table 1).

**Table 1:** Comparison between clinical and laboratory characteristics of the cirrhotic versus noncirrhotic groups.

Variables	General (*n* = 54)	Cirrhotic (*n* = 47)	Noncirrhotic (*n* = 7)	*p* value
Female gender	31 (57.4%)	26 (55.3%)	5 (71.4%)	*p* = 0.685*
Age at PH diagnosis median (Q1–Q3) in years	2.5 (1.1–7.3) *n* = 42	3.0 (0.9–7.5) *n* = 35	1.8 (1.3–4.8)	*p* = 0.956** *r* = 0.01
Age at HPS diagnosis (median (Q1–Q3) in years)	7.8 (4.7–10.8)	7.7 (4.2–11.0)	8.0 (7.6–10.5)	*p* = 0.652** *r* = 0.06
Interval between PH‐HPS diagnosis (median (Q1–Q3) in years)	4.0 (2.3–6.3) *n* = 42	3.6 (2.2–6.3) *n* = 35	4.8 (2.9–6.8)	*p* = 0.161** *r* = 0.22
PaO_2_ (mean ± SD) mmHg	58.2 ± 10.9	58.2 ± 11.1	58.3 ± 10.5	*p* = 0.767** *r* = 0.04
HPS severity				*p* = 0.756*
Moderate HPS	24 (44.5%)	21 (44.7%)	3 (42.8%)	
Severe HPS	20 (37.0%)	18 (38.3%)	2 (28.6%)	
Very severe HPS	10 (18.5%)	8 (17.0%)	2 (28.6%)	
Asymptomatic	24 (44.4%)	20 (42.6%)	4 (57.1%)	*p* = 0.687*
Dyspnea	30 (55.6%)	27 (57.4%)	3 (42.9%)	*p* = 0.687*
Platypnea	1 (1.9%)	1 (2.1%)	0	*p* = 1.000*
Tachypnea	11 (20.4%)	11 (23.4%)	0	*p* = 0.322*
Cyanosis	21 (38.9%)	17 (36.2%)	4 (57.1%)	*p* = 0.411*
Clubbing	35 (64.8%)	31 (66%)	4 (57.1%)	*p* = 0.687*
Palmar erythema	23 (42.6%)	21 (44.7%)	2 (28.6%)	*p* = 0.685*
Spider nevi	15 (27.8%)	15 (31.9%)	0	*p* = 0.171*
Ascites	4 (7.4%)	4 (8.5%)	0	*p* = 1.000*
Orthodeoxia	1 (1.9%)	1 (2.1%)	0	*p* = 1.000*
Encephalopathy	2 (3.7%)	2 (4.3%)	0	*p* = 1.000*
SpO_2_ (mean ± SD) %	88.4 ± 8.9 *n* = 41	88.5 ± 9.1 *n* = 37	87.8 ± 6.7 *n* = 4	*p* = 0.567** *r* = 0.09

Abbreviations: HPS, hepatopulmonary syndrome; PaO_2_, partial pressure of arterial oxygen; PH, portal hypertension; Q1: first quartile; Q3: third quartile; SD, standard deviation; SpO_2_, peripheral oxygen saturation.

* Probability of significance of Fisher's exact test

** Probability of significance of the Mann–Whitney *U* test. *r*: effect size for the nonparametric test.

Patients with cirrhosis and BA were compared with those with other CLD. The median age in the BA group was lower at the time of diagnosing PH (0.8 vs. 5 years; *p* = 0.001; *r* = 0.58) and HPS (5.9 vs. 9.9 years; *p* < 0.001; *r* = 0.51). Other clinical characteristics were similar between the groups, including the severity of HPS (*p* = 0.144), Child‐Pugh classification (*p* = 0.914), and PELD score (*p* = 0.817).

### 3.4 Evolution of HPS and outcomes

The median follow‐up duration was 3 years (0.9–5.5). Home oxygen supplementation was indicated for 53.7% of patients, and the median time between the diagnosis of HPS and the start of oxygen therapy was 34 days (5.5–303.5).

Among the 44 patients (81.5%) indicated to undergo LT, 35 patients underwent LT. Five patients waited for transplantation, one died, and one was lost to follow‐up after LT was indicated. Indication for LT was withdrawn in two cirrhotic patients (Child‐Pugh A5) who exhibited improvement before LT. The administration of immunosuppressants treated the underlying disease in the first patient diagnosed with AIH. The PaO_2_ and A‐aO_2_ gradient were 63.4 mmHg and 35 at the time of diagnosing HPS. The corresponding values at time of the last consultation after approximately 1 year was 102.8 mmHg and −7.8, respectively. The pre‐LT consultation of another patient diagnosed with PFIC 3 and HPS (PaO_2_ 60.5 mmHg/A‐aO_2_ gradient 35/positive CE‐TTE) was postponed owing to the coronavirus pandemic. Measurements taken 1.5 years later revealed PaO_2_ and A‐aO_2_ gradient of 92 mmHg and 2.1, respectively. Tests conducted 2.3 years later revealed spontaneous reversal of hypoxemia (PaO_2_ 82 mmHg/A‐aO_2_ gradient 11.4) and normal CE‐TTE. Thus, therapeutic measures were not implemented.

Ten patients were not considered for LT. Two were lost to follow‐up and one died before undergoing transplant evaluation. Three patients had comorbidities that contraindicated LT including severe neurological disability, pulmonary hypertension, and immunodeficiency. Two patients were not listed due to social issues. One non‐cirrhotic patient with PH due to EHPVO is under evaluation for a Meso‐Rex shunt procedure. Additionally, one cirrhotic patient who was recently diagnosed with HPS is still under evaluation. The frequency of LT indications was significantly higher in the cirrhotic than in the non‐cirrhotic group. No significant differences were observed in other characteristics related to the evolution of the cases (Table 2).

**Table 2:** Comparison between the evolution of hepatopulmonary syndrome in cirrhotic and noncirrhotic patients.

Features	General (*n* = 54)	Cirrhotic (*n* = 47)	Noncirrhotic (*n* = 7)	*p* value
Follow‐up time after HPS diagnosis (median [Q1–Q3] in years)	3.0 (0.9–5.5)	3.2 (0.8–5.6)	3.0 (1.7–3.2)	*p* = 0.671**; *r* = 0.06
Home oxygen therapy	29 (53.7%)	24 (51.1%)	5 (71.4%)	*p* = 1.000*
Time between HPS diagnosis and oxygen therapy initiation (median [Q1–Q3] in days)	34.0 (5.5–303.5)	32.0 (5.3–471.0)	46.0 (6.0–210.0)	*p* = 1.000**; *r* = 0.00
LT indicated	44 (81.5%)	41 (87.2%)	3 (42.8%)	*p* =0.017*
LT performed	35 (79.5%) *n* = 44	34 (82.9%) *n* = 41	1 (33.3%) *n* = 3	*p* = 0.101*
Deaths	10 (18.5%)	10 (21.3%)	0	*p* = 0.322*

Abbreviations: HPS, hepatopulmonary syndrome; LT, liver transplant; Q1, first quartile; Q3, third quartile.

* Probability of significance of Fisher's exact test

** Probability of significance of the Mann–Whitney *U*‐test. *r*: effect size for the nonparametric test.

The median time between the diagnosis of HPS and LT was 6.8 months (3.5–13.3). Most transplantations were performed using living donors (85.7%). The median time on mechanical ventilation (MV) post‐LT was 1 day.^1^, ^2^, ^3^ The mean SpO_2_ 30 days post‐LT was 96.6 ± 2.7%. PaO_2_ (*p* = 0.339), severity of HPS (*p* = 0.091), response to the hyperoxia test (*p* = 0.068), mean hemoglobin level (*p* = 0.759), and severity of cirrhosis (PELD, *p* = 0.263; MELD, *p* = 0.91; Child‐Pugh, *p* = 0.112) at the time of diagnosing HPS had no effect on the duration of MV post‐LT.

**Figure 1:**
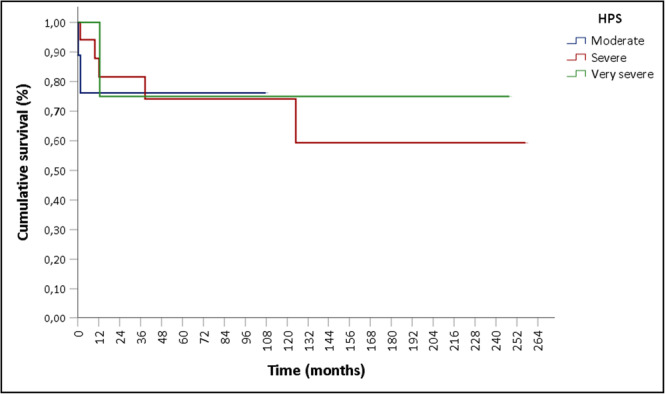
Post–liver transplant survival by HPS severity classification. HPS, hepatopulmonary syndrome.

The post‐transplant survival rates for the overall group were 90,3% and 84,6% at 2 months and 1 year, respectively. No differences were observed between the survival rates of the groups according to the differences in the HPS severity classifications (log‐rank test *χ* ^2^_(2)_ =0.479; *p* = 0.787) (Figure 1).

Ten patients in the cirrhosis group died. Eight patients died post‐LT, with three deaths occurring within 60 days of surgery (10 days post‐LT owing to diffuse gastric hemorrhage, 43 days post‐LT owing to septic shock, pulmonary edema, and pancreatitis, and 47 days post‐LT owing to arrhythmia during perioperative retransplantation). The remaining five patients died 10 months, 1 year, 1 year, 3 years, and 11 years post‐LT owing to complications unrelated to HPS. Outcomes of the 54 patients can be found in the Figure S1.

### 3.5 Laboratory changes from underlying disease diagnosis to HPS diagnosis

Biochemical and liver function tests (aspartate aminotransferase, alanine aminotransferase, gamma‐glutamyl transferase, and bilirubin levels; prothrombin time; and activated partial thromboplastin time) did not indicate a worsening tendency compared with that at the time of diagnosing the underlying disease and HPS. Analysis of data from 26 patients with hemogram results available at three key time points during the follow‐up period (at the time of diagnosing the underlying disease, 1 year before diagnosing HPS, and at the time of diagnosing HPS), revealed an increase in the mean hemoglobin (Hb) levels and a decrease in the median leukocyte and platelet counts (Table 3).

**Table 3:** Hemogram data throughout the follow‐up period.

Laboratory data	Underlying disease diagnosis (1)	1 year before HPS diagnosis (2)	HPS diagnosis (3)	*p* value
Hb (mean ± SD) g/dL	11.0 ± 2.4	11.4 ± 2.0	12.8 ± 1.9	*p* = 0.009; *r *= 0.72 (1 = 2) < 3
Leucocytes (median [Q1–Q3])	9.235/µL (5.715–12.815)	4.515/µL (3.775–5.873)	4.275/µL (3.560–4.705)	*p* < 0.001; *r* = 2.01 1 > (2 = 3)
Platelets (median [Q1–Q3])	204.000/µL (123.500–443.750)	85.500/µL (68.000–120.500)	90.500/µL (69.750–125.750)	*p* < 0.001; *r* = 2.36 1 > (2 = 3)

*Note*: *p*, Probability of significance of the Friedman test (*Z* to the test statistics). *r*: Effect size for the nonparametric test.

Abbreviations: Hb, hemoglobin; HPS, hepatopulmonary syndrome; Q1, first quartile; Q3, third quartile; SD, standard deviation.

## 4 DISCUSSION

HPS is a complication of PH with poor prognosis. Few studies evaluated HPS in pediatric populations, with most studies being single‐center investigations with small sample sizes. This multicenter study provides a longitudinal follow‐up of a significant cohort of pediatric patients with HPS and presents data from different stages of their clinical history.

Patients with cirrhosis‐related PH were predominant in the sample. This finding may have been influenced by sampling bias; however, as most participating centers treated patients referred for LT, it is consistent with the findings of previous studies that revealed a higher prevalence of HPS in cirrhotic compared with non‐cirrhotic patients.^4^, ^8^, ^9^ Furthermore, the proportion of children with BA as an underlying disease was higher, and the median age at the time of diagnosing HPS was lower in this group, reinforcing the rapid and progressive development of cirrhosis and PH among these patients.^10^, ^11^

At the time of diagnosing HPS, 44.4% of the patients were asymptomatic. The mean PaO_2_ was higher in these patients, indicating that HPS results from the progressive evolution of pulmonary vascular alterations over time. The present study revealed that the onset of symptoms is related to the worsening of hypoxemia, reinforcing the fact that the disease is silent in its initial stages.^12^, ^13^, ^14^ Consistent with the findings of previous reports, digital clubbing, palmar erythema, and cyanosis were the most common findings on physical examination.^14^, ^15^, ^16^, ^17^, ^18^, ^19^

The ILTS guidelines recommend screening for HPS by assessing SpO_2_ levels ^3^ based on data demonstrating 100% sensitivity of SpO_2_ < 96% for identifying adult patients with PaO_2_ <70 mmHg.^20^ However, the accuracy of this method is not the same in pediatric populations.^5^, ^9^ The present study emphasizes the limitation of pulse oximetry for HPS screening in children, given that eight (14.8%) patients with SpO_2_ of ≥96% would have been missed. Seven of these patients had a PaO_2_ of <70 mmHg. Well‐established screening strategies for HPS remain to be established.

The present study revealed no correlation between the severity of HPS and cirrhosis as assessed by the Child‐Pugh, PELD, and MELD scores. Previous studies have reported divergent findings on this topic.^4^, ^5^, ^7^, ^10^, ^17^, ^19^, ^21^ Similarly, laboratory results have been inconsistent in literature.^4^, ^7^, ^8^, ^16^ In the present study, the biochemical parameters or liver function values exhibited no consistent pattern over the follow‐up period. Regarding hemogram data, some expected changes were observed, such as a decrease in platelet and leukocyte counts owing to the worsening of hypersplenism. However, in the paired analysis, mean hemoglobin levels increased between the baseline and the development of HPS. This finding contradicts expectations for patients with progressing cirrhosis and hypersplenism and may represent a compensatory response to chronic hypoxemia. The hemoglobin levels in HPS have previously been investigated as a predictive factor for increased risk of hepatic artery thrombosis post‐LT.^15^, ^16^ However, no longitudinal data have demonstrated an increase in hemoglobin levels relative to the baseline as a potential indicator of the onset of hypoxemia in patients with PH. To the best of our knowledge, this is the first study to report these findings.

Most patients who underwent LT received organs from living donors. The median interval between the diagnosis of HPS and LT was 6.8 months, a period typically required for donor and recipient preparation, as well as logistical arrangements.

The post‐LT survival rates were 90.3% and 84.6% at 2 months and 1 year, respectively, in the present study. Three Brazilian studies reported an overall 1‐year post‐LT survival rates of 90.3%–93% in pediatric recipients.^22^, ^23^, ^24^ Some studies compared the post‐LT survival of patients with and without HPS and reported similar or slightly lower values in patients with HPS.^5^, ^15^, ^25^ The 1‐year post‐LT survival rate in the present study was slightly lower than the overall rate in Brazil, which may be attributed to the multicenter design and long follow‐up period. Furthermore, some new cases did not have sufficient post‐LT follow‐up for inclusion in the survival analysis and were subsequently censored.

The severity of HPS was not correlated with worse outcomes in the present study. Previous studies have revealed that more severe hypoxemia was associated with higher rates of postoperative complications. However, this did not lead to higher mortality.^25^, ^26^, ^27^ This finding contrasts with the findings in adult populations, wherein more severe hypoxemia is associated with higher mortality.^28^, ^29^

This discrepancy between adult and pediatric populations raises concerns regarding the criteria used by organ allocation systems to prioritize HPS patients on the LT waiting list. Priority is given to those with PaO_2_ <60 mmHg owing to the higher mortality rate observed in adults with severe hypoxemia.^3^, ^21^ Further research is required to refine these criteria, given that this correlation does not apply to pediatric patients.

The risk factors associated with mortality in patients with HPS could not be evaluated owing to the small number of events. The present study indicates the encouraging survival rates post‐LT as a key factor when considering these patients for the procedure. The availability of living donor transplants and data indicating higher rates of postoperative complications in patients with more severe hypoxemia ^26^, ^27^ emphasizes that waiting for PaO_2_ < 60 mmHg before considering LT is not mandatory.

A mean SpO_2_ measurement of >96% on room air was observed 30 days post‐LT, consistent with the findings of several pediatric studies reporting HPS resolution rates approaching 100% post‐LT.^5^, ^15^, ^16^

This case series also documented two cases of improvement in HPS without LT. Similar cases have been reported in the literature, with improvement in the underlying liver disease being observed in most cases.^10^, ^30^, ^31^ However, the patient with cirrhosis who experienced complete resolution of HPS without any concurrent improvement in the baseline liver condition represents a novel finding not previously described.

The present study is a retrospective study with limitations related to incomplete data from medical record reviews; however, it presents a multicenter case series involving leading reference centers for pediatric LT in Brazil that makes a significant contribution to the field of pediatric hepatology.

## 5 CONCLUSION

This study reinforces data that demonstrates that HPS is silent during its initial stages. SpO_2_ may not be a reliable screening test in pediatric population. An increase in the hemoglobin level in a patient with PH may be related to hypoxemia, indicating the onset of this complication. Data obtained at the time of diagnosing HPS (PaO_2_, hyperoxia test, Hb, and cirrhosis severity) were not correlated with the post‐LT MV time. The severity of hypoxemia at the time of diagnosing HPS does not affect post‐LT survival.

## CONFLICT OF INTEREST STATEMENT

The authors declare no conflicts of interest.

## Supporting information

Revised ‐ Supplemental Figure 1.
