# Supplementary material for: Hepatopulmonary syndrome in children and adolescents with portal hypertension in Brazil: A multicenter study
Source: J Pediatr Gastroenterol Nutr. 2025 Dec 5;82(2):366–73. doi: 10.1002/jpn3.70306 (PMC12864179; doi:10.1002/jpn3.70306)
Supplement: Supplementary file 1 — Revised ‐ Supplemental Figure 1. [file JPN3-82-366-s001.docx]

**Supplemental Digital Content (SDC)**

**Supplemental Figure 1:** Outcomes of patients with hepatopulmonary syndrome.

HPS: hepatopulmonary syndrome; LT: liver transplant.
